# Two-Dimensional Polyacrylamide Gel Electrophoresis for Metalloprotein Analysis Based on Differential Chemical Structure Recognition by CBB Dye

**DOI:** 10.1038/s41598-019-46955-6

**Published:** 2019-07-22

**Authors:** Junko Ishikawa, Akinori Maeshima, Allyson Mellinger, Anne Durand, Marie-Line Bourbon, Daichi Higo, Christa L. Colyer, Masami Shibukawa, Soufian Ouchane, Shingo Saito

**Affiliations:** 1 Graduate School of Science and Engineering, Saitama University, 255 Shimo-Okubo, Sakura-ku, Saitama 338-8570 Japan; 2 Department of Chemistry, Wake Forest University, Winston-Salem, North Carolina 27109 United States; 3 Institute for Integrative Biology of the Cell (I2BC), CEA, CNRS, Univ. Paris-Sud, Universitè Paris-Saclay, 91198 Gif sur Yvette cedex, France

**Keywords:** Metalloproteins, Metalloproteins, Bioanalytical chemistry, Metalloproteins, Metals

## Abstract

In an effort to develop an analytical method capable of finding new metalloproteins, this is the first report of a new diagonal gel electrophoresis method to isolate and identify metalloproteins, based on the molecular recognition of holo- and apo-metalloproteins (metalbound and -free forms, respectively) by CBB G-250 dye and employing metal ion contaminant sweeping-blue native-polyacrylamide gel electrophoresis (MICS-BN-PAGE). The difference in electrophoretic mobilities between holo- and apo-forms was exaggerated as a result of interactions between the metalloproteins and the dye with no metal ion dissociation. The different binding modes of proteins with CBB G-250 dye, primarily related to hydrogen bonding, were confirmed by capillary zone electrophoresis (CZE) and molecular docking simulations. Due to in-gel holo/apo conversion between the first and second dimensions of PAGE, holo-metalloproteins in the original sample were completely isolated as spots off the diagonal line in the second dimension of PAGE. To prove the high efficiency of this method for metalloprotein analysis, we successfully identified a copper-binding protein from a total bacterial soluble extract for the first time.

## Introduction

Identifying which proteins bind (or don’t bind) to which metal ions in raw biological samples is key to understanding many biological processes involving metal ions, since it is known that one-third of proteins are metalloproteins and most of these possess important regulatory or catalytic functions and structural roles^1–4^. In addition, it has already been revealed that many metalloproteins are involved in serious diseases (including Wilson disease, Parkinson’s disease, Alzheimer’s disease and cancer)^5–8^. Thus, to reveal metal-binding state, structure and distribution of metalloproteins is of importance by means of various chemical approaches. Haraguchi^2^ and Szpunar^9^ individually proposed “metallomics”, which is the total analysis of chemical species involving complexation with metal ions, especially metalloprotein (metal-bound protein) species in biological samples. Since then, researchers, especially chemists in the field of separation science, have developed many analytical methods for metalloprotein determination^1,10^. These methods were largely designed for the detection of metal ion distribution in metalloproteins using ordinary separation strategies for proteins, and they may be categorized into two types: one using liquid chromatographic (LC) separations coupled with instrumental elemental analysis such as inductively-coupled plasma-mass spectrometry (ICP-MS)^3,11^; and the other using polyacrylamide gel electrophoresis (PAGE) coupled with laser-ablation (LA)-ICP-MS^12,13^. A number of metalloprotein studies employing these methods over the last two decades have proven the importance of metalloprotein analysis in the biological field due to the role of metal-based species in the control of many biological phenomena^4,6^. However, no separation method was specifically aimed at the selective isolation of metalloproteins, thus making “metallomics” a yet-to-be-realized domain.

These metalloprotein methods can suffer from the dissociation of metal ions from holo (metal-bound)- to apo (metal-free)-metalloprotein upon the addition of denaturing agents^14,15^, along with serious contamination of metal ions in the separation field^3,11,15–17^. A negative consequence of metal ion dissociation is the fact that holo-metalloproteins may be misidentified as apo-metalloproteins. To solve this problem, some PAGE-based methods have been proposed as effective ways for separation without dissociation of metal ions under weak denaturing or native conditions, including: blue native (BN)-^15–17^, native SDS-^14^ and quantitative preparative native continuous (QPNC)-PAGE^18^. In terms of LC methods, few options to avoid metal dissociation have been reported, with the exception of non-denaturing size-exclusion chromatography (SEC)^19,20^. Such non-denaturing methods, however, do not accomplish contaminant-free analysis. Serious contamination by metal ions at ppb levels can originate during separation processes from instruments (for example, glass plates and electrodes for PAGE, and tubing and sintered filters for LC) and reagents (for example, gel monomers and elution agents at high concentrations). Even for many LC-ICP-MS methods, this contamination problem is unavoidable since the mobile phase (possibly with low ppb levels of metal ion contamination) is continuously delivered during the elution. This must cause misidentification of apo-metalloprotein as holo-metalloprotein due to the misuptake of contaminant metal ions. In addition, the complete separation of metalloproteins from all other protein species in biological samples to identify metal-binding species by mass spectrometry is generally impossible by means of SEC and PAGE. Thus, we identified the remaining need for a selective isolation and identification method excluding metal contaminants as a total analysis system for holo-metalloproteins, which is developed herein.

To address the issue of contaminant metal ions, we have previously studied thermodynamically and kinetically stable metal chelates to exhaustively remove trace contaminant metal ions from the separation field in PAGE^21^. In this method, which we have called metal ion contaminant sweeping-blue native-PAGE (MICS-BN-PAGE), the cationic TPEN (*N,N,N*′,*N*′-tetrakis (2-pyridylmethyl) ethylene-diamine) complexes and anionic EDTA complexes formed with contaminant metal ions migrate towards the cathodic and anodic directions, respectively. By this method, the electrophoretic separation of biological samples is possible without their proteins encountering any doubly- and triply-charged contaminant metal ions (since the concentrations of such contaminants are decreased to lower than ppt levels). This effectively avoids misidentification of apo-metalloproteins as holo-metalloproteins. Furthermore, MICS-BN-PAGE also avoids the possibility of metal-exchange reactions of holo-metalloproteins with contaminant metal ions M′’^2+^ (e.g. M^2+^-metalloprotein + M′^2+^ → M′^2+^-metalloprotein + M^2+^), which lead to the misidentification of metalloprotein species by conventional methods^21^. Still, the most difficult challenge remains, which is to isolate metalloproteins while simultaneously ensuring no dissociation of metal ions and the absence of contaminant metal ions in the separation field.

Our starting point for selective isolation of metalloproteins in the present work was by enabling their molecular recognition, which provided for their different electrophoretic mobilities. Such recognition was intended to electrophoretically differentiate between holo- and apo-metalloproteins without dissociation of metal ions bound to the holo-form (see Results and Discussion). Building upon this finding led us to a new methodology as described herein: the holo/apo conversion (HAC)-2D MICS-BN-PAGE methodology for the selective isolation of holo-metalloproteins. In this paper, we present not only the concept of HAC-2D MICS-BN-PAGE for identification of metalloproteins, but also the mechanism of electrophoretic molecular recognition of holo-/apo-protein forms, and using this novel technology we successfully isolated and identified a bacterial copper binding protein from a total soluble protein sample.

## Results and Discussion

### Differential migration of holo- and apo-metalloproteins by MICS-BN-PAGE

**Figure 1:**
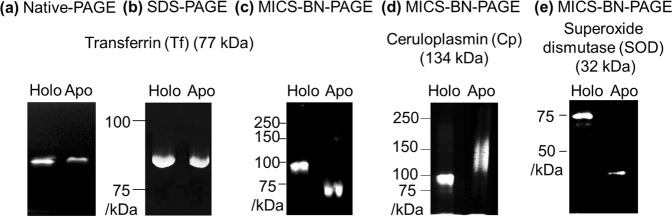
Electropherograms of standard metalloproteins holo- and apo-transferrin (Tf) by Native-PAGE (**a**), by SDS-PAGE (**b**) and by MICS-BN-PAGE (**c**); ceruloplasmin (Cp) by MICS-BN-PAGE (**d**); and superoxide dismutase (SOD) by MICS-BN-PAGE (**e**).

While no separation of holo- (Fe_2_-transferrin (Tf)) and apo-Tf was observed in conventional 1D native (CBB G-250 free)-PAGE (Fig. 1a) and SDS-PAGE (Fig. 1b), interestingly, we found that holo- and apo-Tf are completely separated by means of MICS-BN-PAGE (Fig. 1c) (it should be noted that two bands were observed for a “pure” apo-Tf sample using BN-PAGE without MICS mode due to metal ion contamination; Supplementary Fig. S1). In SDS-PAGE, this is probably due to the dissociation of metal ions from holo-forms occurring under strong denaturing conditions^14,15^ (data not shown). This fact suggests that the electrophoretic recognition between holo- and apo-forms is not available for conventional PAGE methods. These results imply that specific weak denaturing agents, like CBB-G 250 employed in MICS-BN-PAGE, recognize the difference between holo- and apo-metalloproteins to enhance the separation, in addition to avoiding metal dissociation.

Different migration behaviours for holo- and apo-forms were also observed for ceruloplasmin (Cp) bound with Cu^2+^ (Cu-Cp) and superoxide dismutase (SOD) bound with Cu^2+^ and Zn^2+^ (Cu/Zn-SOD) by the MICS-BN-PAGE method (Fig. 1d,e). Apo-forms migrated to positions in close correspondence with their accurate molecular weights in MICS-BN-PAGE (Fig. 1: Tf, 77 kDa; Cp, 134 kDa; SOD, 32 kDa), which is useful when identifying the proteins. As described in the Introduction section, these findings led us to develop the holo/apo conversion (HAC)-2D MICS-BN-PAGE methodology for selective isolation of holo-metalloproteins.

### Concept of holo/apo conversion 2D MICS-BN-PAGE

**Figure 2:**
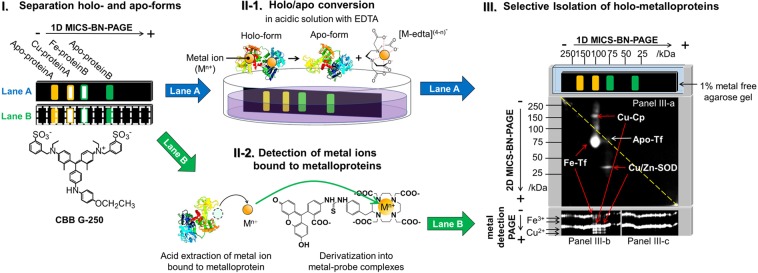
Concept of HAC-2D MICS-BN-PAGE/metal detection PAGE methodology. Panel I: 1D MICS-BN-PAGE in two lanes. Lane A was subjected to the in-gel holo/apo conversion procedure (as in Panel II-1), and then Lane A was subjected to 2D MICS-BN-PAGE after holo/apo conversion (as in Panel III). Lane B was subjected to Cu^2+^ and Fe^3+^ detection PAGE (as in Panel II-2). The 2D electropherogram is for a mixture of metalloproteins (holo-Tf, apo-Tf, holo-Cp and holo-SOD), in which holo-metalloproteins in the original sample were isolated as spots off the diagonal line (the dotted yellow line in Panel III). Full-size versions of electropherograms represented in Fig. 2 are depicted in Fig. S2.

Our new 2D PAGE method based on the differential migration between holo- and apo-metalloproteins, begins with a MICS-BN-PAGE stage conducted in two lanes (lanes A and B in Fig. 2, panel I) to allow for the separation of holo- and apo-metalloproteins from a sample containing both metalloprotein forms. The two lanes are then subjected to two different treatments after the initial MICS-BN-PAGE separation: Lane A is subjected to a second MICS-BN-PAGE stage but only after undergoing a holo/apo conversion treatment (Fig. 2 panel II-1); while Lane B is delivered to a metal detection PAGE stage to determine the metal ions associated with the separated proteins (Fig. 2 panel II-2). The treatment applied to Lane B has been previously validated. For example, the determination of some heavy metal ions (Cu^2+^, Fe^3+^, Co^3+^, Ni^2+^, Mn^2+^ and Cd^2+^) in small volume (µL) samples at ppt and sub-ppb levels by this PAGE method without the use of a clean room have been reported^21^. Furthermore, this tandem 1D MICS-BN-PAGE/metal detection PAGE methodology revealed the precise distribution of Cu^2+^ in human serum even for weakly albumin-bound (exchangeable) Cu^2+^ ^21^, and suggested that the periplasmic *Rubrivivax gelatinosus* CopI protein highly involved in copper resistance is a copper-binding protein^22^. Still, to completely identify metal-bound proteins in a complex protein sample is difficult due to co-migrating proteins. For example, it has not been fully proven that CopI is a copper-binding protein in *R. gelatinosus*, although its sequence has three putative Cu-binding sites: (i) a type 1 copper center present in many cupredoxins such as azurin and plastocyanin, (ii) a Histidine-rich N-terminus sequence and (iii) a Histidine/Methionine-rich sequence. CopI-related proteins are present in many bacteria but are not widely conserved; for example, it is absent from *Escherichia coli*^22^. So far, only the proteins of *R. gelatinosus* and *Vibrio cholerae* have been studied and are involved in Cu resistance^22,23^. In *R. gelatinosus*, CopI protein is highly expressed in the presence of Cu^2+^; however, the Cu-detoxification mechanism is still unknown. Thus, a method for isolation of metalloproteins from all other proteins co-existing in complex biological samples is necessary.

To overcome this limitation, the information provided by the 1D MICS-BN-PAGE/metal detection PAGE analysis of Lane B is augmented by subjecting Lane A to a second dimension of MICS-BN-PAGE after holo/apo conversion. To effect this analysis, Lane A is first soaked in an acidic, metal chelator solution (see Methods section and Supplementary for the detailed conditions) for holo/apo conversion (Fig. 2 panel II-1). In so doing, the holo-metalloproteins are derivatized into metal-free apo-metalloproteins in the gel of the first PAGE dimension. The treated Lane A is then delivered to the second MICS-BN-PAGE stage with the aid of a metal-free agarose gel (see Supplementary Information). In the second MICS-BN-PAGE stage (Fig. 2, panel III), apo-metalloproteins and non-metalloproteins from the original sample migrate on the diagonal line, since the migration of these species in the second PAGE dimension is completely identical to their migration in the first PAGE dimension. On the other hand, holo-metalloproteins from the original sample migrate different distances in the first and second MICS-BN-PAGE dimensions, since these proteins migrate as their holo-forms in the first dimension and as their apo-forms in the second dimension (and since MICS-BN-PAGE results in a different electrophoretic mobility for holo- and apo-forms of metalloproteins; *vide infra*). Thus, only holo-metalloproteins from the original sample migrate off the diagonal line in the second dimension, to be identified by MALDI-TOF MS without any additional protein purification. The types and quantities of metal ions bound with metalloproteins in the original sample solution (those isolated as off-diagonal spots), can be determined by metal detection PAGE of Lane B, as described above. Hence, information pertaining to the identification and distribution of metalloprotein species, along with the identities and concentrations of metal ions they bind, is accessible from this new HAC-2D MICS-BN-PAGE/metal detection PAGE methodology.

### Selective isolation of metalloproteins in HAC-2D MICS-BN-PAGE

For proof-of-concept, HAC-2D MICS-BN-PAGE/metal detection PAGE was demonstrated for a sample mixture containing holo-Tf, apo-Tf, holo-Cp and holo-SOD protein standards (resulting in the 2D electropherogram in Fig. 2, panel III), and also for a human serum sample (Supplementary Fig. S3). Spots of holo-metalloproteins that migrated off the diagonal line were successfully observed for the mixed protein sample. In addition, Fe^3+^ was detected at the position of holo-Tf, and Cu^2+^ at the positions of holo-Cp and holo-SOD, in the first MICS-BN-PAGE stage using metal detection PAGE. If no holo/apo conversion is performed, all the proteins migrate on the diagonal line (Supplementary Fig. S5). For a human serum sample, holo-Tf and holo-Cp were successfully detected (Supplementary Fig. S3). Thus, it was concluded that HAC-2D MICS-BN-PAGE/metal detection PAGE is very effective for the isolation of holo-metalloproteins and the identification of metal ions originally bound to metalloproteins in a protein mixture.

**Figure 3:**
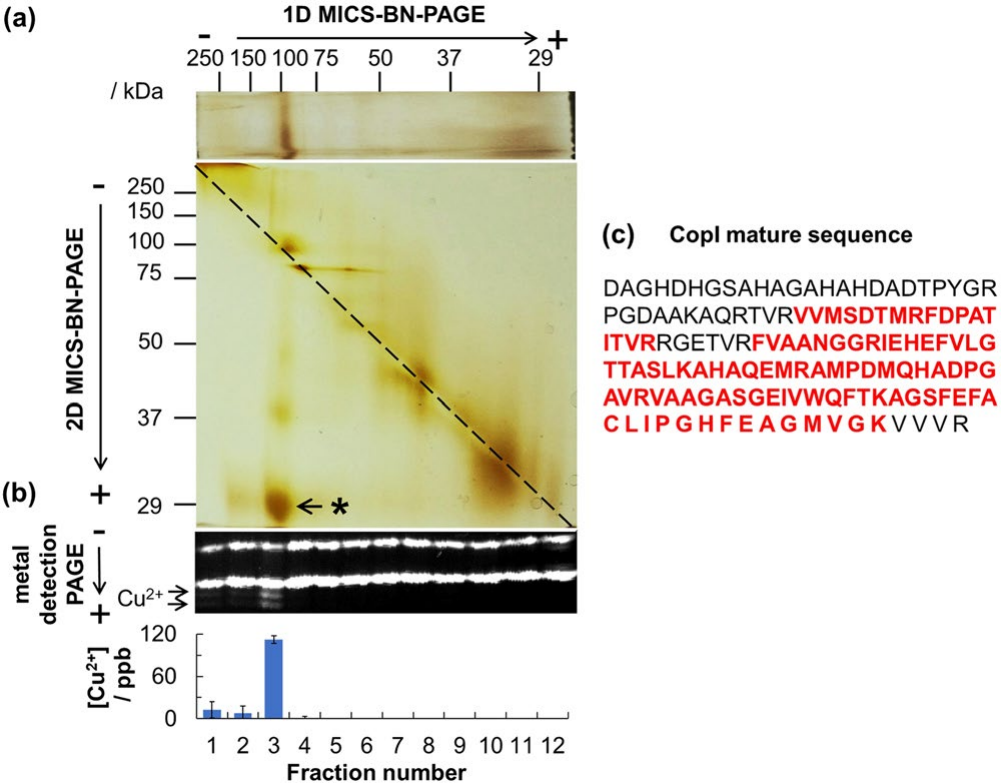
HAC-2D MICS-BN-PAGE employing silver staining (**a**), with Cu^2+^ detection PAGE (**b**), of a soluble fraction obtained from *R. gelatinosus* cells grown in the presence of 1.2 mM Cu^2+^ (total protein: 31.5 µg). The off-diagonal spot indicated by the asterisk in (**a**) was subjected to MALDI-TOF MS (Supplementary Fig. S7), identifying the sequences shown in red font as part of the CopI mature sequence (**c**). Full-size versions of electropherograms represented in Fig. 3 are depicted in Supplementary Fig. S8.

Finally, the HAC-2D MICS-BN-PAGE method was applied to a total bacterial soluble protein fraction obtained from *R. gelatinosus* cells grown in the presence of 1.2 mM Cu^2+^, expressing CopI protein. This represents a biological protein sample. For this sample, a spot off the diagonal line was found at the position of 100 kDa and 29 kDa in the first and second PAGE, respectively (Fig. 3a). A high concentration of Cu^2+^ was observed at the position of 97–139 kDa in the first MICS-BN-PAGE (Fig. 3b). Consequently, it was concluded that the protein in this spot has Cu ion bound to it. The spot was cut and then subsequently analyzed by MALDI-TOF MS. Peptides corresponding to CopI were identified (Fig. 3c, Supplementary Fig. S7 and Table S1). In addition, when this gel spot was run on SDS-PAGE, a single band of CopI protein was observed (data not shown). Another spot off of the diagonal line was observed at around 40 kDa (above the 29 kDa CopI spot in the second dimension (Fig. 3a)). It was confirmed by MALDI-TOF MS that this spot also originated from CopI (and was probably a dimer of CopI according to the molecular weight). Thus, a metalloprotein could be specifically isolated from a complex protein mixture while also identifying its bound metal. This analysis by HAC-2D MICS-BN-PAGE definitely proved that CopI is a copper binding-protein, and interestingly, that this property could be related to the protein’s Cu-detoxifying function in the bacterial periplasm. Further quantitative analysis revealed the stoichiometry of Cu ion to CopI as 1:1 (based upon the concentrations of the bound Cu ion (1.05 µM) and of the holo-CopI protein (0.95 µM) as determined by CBB R-250 staining). This is in total agreement with other experimental results, which found a Cu:CopI stoichiometry of 1:1.2 using ICP-MS with pure CopI (Durand *et al*., unpublished data). This result suggests that our method is also useful for investigations of metalloprotein stoichiometries.

### Capillary electrophoresis experiments to investigate molecular recognition modes

**Figure 4:**
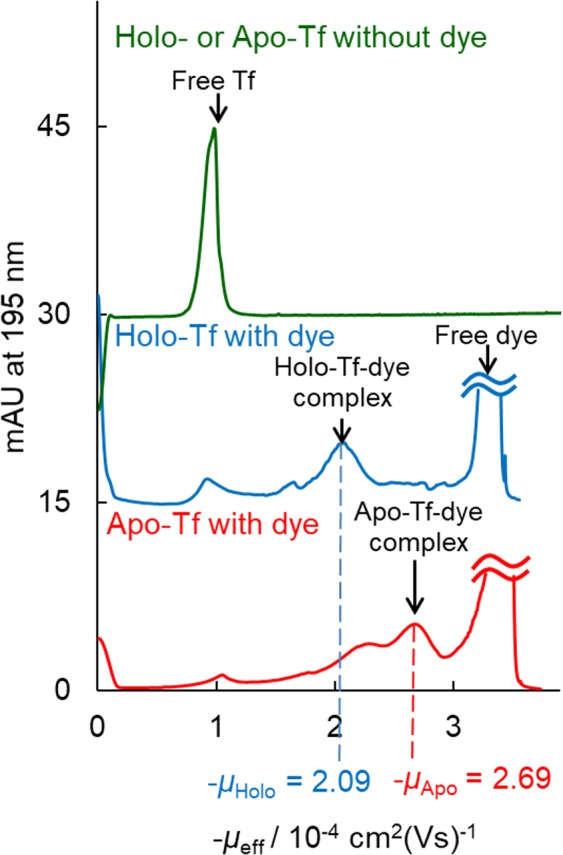
Typical electropherograms from CZE experiments for: 10 µM holo- or apo-Tf without CBB G-250 (upper green trace); and mixtures containing 5 mM CBB G-250 dye with 10 µM holo-Tf (middle blue trace) or with 10 µM apo-Tf (lower red trace).

The resolution enhancement between holo- and apo-forms in MICS-PAGE assisted with CBB dye (Fig. 1c–e) is an important key to our methodology, and, interestingly, this molecular recognition seems to originate from different numbers of CBB G-250 dye molecules binding to each of the holo- vs. apo-forms of metalloproteins. This, in turn, likely results in different effective charges, and/or induced steric structures, for the dye-protein complexes. Both of these effects would impact electrophoretic mobility. To obtain deeper insight regarding the recognition mechanism of CBB G-250 dye binding towards holo- and apo-Tf, CE experiments were conducted, along with docking simulations using the AutoDock Vina program^24,25^ (*vide infra*). CZE is conducted in free aqueous solution without a gel matrix, and so a molecular sieving effect need not be considered in the simulations. The electrophoretic mobility of holo-Tf measured by CZE was obviously different from that of apo-Tf with the addition of the CBB dye (Fig. 4), while these proteins had identical mobilities in the absence of the dye. This fact strongly suggests that the number of dye molecules bound with holo- and apo-metalloproteins is different. In CZE, the migration of the protein-dye complex would be predominantly affected by the effective charge of the complex, which, in turn, would be affected by the number of singly-charged anionic CBB dye molecules bound to the protein.

Traditionally, the electrophoretic mobility of a protein in free solution is represented by the Henry equation, as shown in equation (1):

1 $$\mu =\frac{Q}{4\pi \eta R(1+kR)}H(kR)$$

Here, *Q*, *η*, *R* and *k* represent the net charge, the solution viscosity, the radius of the protein, and the inverse Debye length of electrolyte solution. The Henry function, *H*, increases monotonically from 2/3 to 1. Strictly speaking, this formula applies exclusively to a spherical molecule with a centrosymmetric charge distribution, although there are some discussions to modify the Henry equation to apply to non-spherical proteins with complex charge distribution (including dipole and quadrupole)^26^. In our case, holo- and apo-Tf molecules are deformed spheres of very similar size (resembling spheroids with long and short axis lengths of approximately 92 and 60 Å (holo-Tf), and 93 and 68 Å (apo-Tf), respectively) (Supplementary Fig. S9). Additionally, the dipole and quadrupole are expected to have only a minor effect on mobility since the CBB G-250 molecules bound to holo-/apo-Tf are not localized, as shown in Supplementary Fig. S9 (see below). The results of precise calculations by Kim *et al*.^26^ show that the electrophoretic mobility of a spheroidal protein is not significantly affected by a quadrupole in solutions of low ionic strength (*I* < 0.01M), and the ionic strength of the separation buffer in our CZE experiments was similarly low (*I* = 0.013M).

Thus, the ratio of electrophoretic mobilities of apo- and holo-Tf complexed with CBB G-250 dye (namely, *μ*_*Apo*_/*μ*_*Holo*_), is attributed to the ratio of the net charges of each complex (namely, *Q*_*Apo*_/*Q*_*Holo*_), which can be easily derived from the Henry equation. The negative net charges of Tf-CBB G-250 complexes mainly originate from the binding number of the dye. Consequently, the value of *μ*_*Apo*_/*μ*_*Holo*_ also gives the ratio of the dye numbers (namely, *N*_*Apo*_/*N*_*Holo*_). Experimentally, the ratio of electrophoretic mobilities of the two forms of Tf (*μ*_*Apo*_/*μ*_*Holo*_ = (−2.69 × 10^−4^)/(−2.09 × 10^−4^)) was found to be 1.29 by CZE (Fig. 4), which corresponds to the ratio of effective electric charge, provided the sizes of both forms of Tf are similar. Hence, the ratio of electrophoretic mobilities should agree with the ratio of dye molecules bound. However, further discussion of the relationship between electrophoretic mobility and protein shape for other metalloproteins is needed in cases when holo- and apo-protein shapes differ (as for Cp).

### Molecular docking simulation experiments to investigate molecular recognition modes

**Figure 5:**
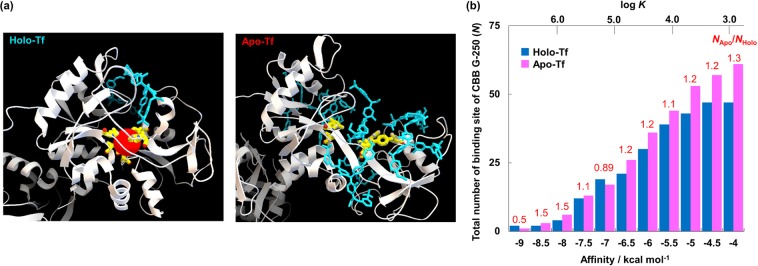
Results from AutoDock Vina calculations: the Fe-binding site structures for holo-Tf (left) and apo-Tf (right) complexes with CBB-G 250 dye (**a**); the dye-binding number cumulative frequency distribution as a function of binding energy for holo-Tf (blue) and apo-Tf (pink) (**b**). Blue, red and yellow atoms in (**a**) represent CBB G-250, Fe^3+^ and amino acid residues bound with Fe^3+^, respectively.

Dye binding was determined by flexible molecular docking simulations by AutoDock Vina^27^, designed to exhaustively search for binding sites and to calculate their free energies (Fig. 5 and Supplementary Fig. S9). Recently, Wang *et al*.^25^ reported that AutoDock Vina has a high scoring power and intermediate sampling power relative to other widely-used docking programs. According to these simulations, greater numbers of CBB G-250 dye molecules bound to vacant Fe-binding sites in apo-Tf compared to holo-Tf (Fig. 5a) were observed. Based on these Autodock Vina simulations, the ratio of the dye-binding number *N*_*Apo*_/*N*_*Holo*_ was found to be 1.23, with a binding energy <−6.5 kcal/mol (corresponding to *K*~10^4.4^ M^−1^) (Fig. 5b for the dye-binding number cumulative frequency distribution as a function of binding energy), which is in good agreement with our CZE results, discussed previously (*N*_*Apo*_/*N*_*Holo*_ = 1.29). Quantitative binding is presumed with the addition of the dye at mM levels (over 95% of binding sites interact with the dye when log *K* is 4.4 or larger), as in these experiments.

The binding number of dye molecules with holo-Tf was also investigated by CZE experiments (data not shown), as judged from the decrease of free dye peak for a 1 mM CBB G-250 dye sample with and without the addition of 10 µM holo-Tf. Since the binding number was measured to be >18, the binding number of *N*_*Holo*_ was presumed to be >20 in the MICS-BN-PAGE experiments (with 3.0 mM dye added). This binding number of dye molecules (>20) from CZE experiments is in good agreement with the number of bound dye molecules (*N* = 21) from molecular docking simulations that would give rise to the predicted affinity of −6.5 kcal/mol, and so our results are self-consistent.

It is seen from these molecular docking simulations and CZE experiments that CBB G-250 dye recognizes the different chemical structures of holo- and apo-metalloproteins to yield different *µ* values. More detailed observations of the simulated binding modes also suggest that the CBB G-250 molecule interacts with amino acid residues accessible due to the more open (unfolded) apo-Tf structure compared to the more closed (folded) holo-Tf structure, while the dye shows no binding directly with Fe-binding amino acid residues (Asp392, Tyr429, Tyr517 and His 585). Thus, it is implied that the dye recognizes small differences between the folded and unfolded chemical structures of metalloproteins, which are induced by the binding or dissociation of metal ions. Such small differences cannot be identified by any other conventional separation methods.

Amino acid residues contacting the dye molecule in each dye-binding site in the simulation (for example, Supplementary Fig. S10) were categorized according to their dominant nature: hydrophobic, hydrophilic, or electrostatic charge (as summarized in Supplementary Table S2 and Table S3). According to the results, the populations of each type of amino acid interacting with dye molecules showed no difference between holo- and apo-protein forms (26–27% for hydrophobic, 37–40% for hydrophilic, 33–35% for charged residues in each form). However, the number of hydrogen bonds in apo-Tf (33 total; 1.2 per binding site) was significantly larger than in holo-Tf (19 total; 0.9 per binding site). When focusing on the eight binding sites in apo-Tf that were not present in holo-Tf (Supplementary Table S3), the number of H-bonds per site was found to be 2.1. These results strongly suggest that changes in hydrogen bond interactions are primarily responsible for the differentiation between holo- and apo-Tf forms, while further investigation would be needed to understand the complete mechanism.

## Conclusion

In this paper, a new technique has been successfully developed, to allow for both the total analysis of metalloproteins and the selective isolation of holo-metalloproteins alone from mixtures. This technique involves protein isolation by HAC-2D MICS-BN-PAGE based on molecular recognition by CBB-G 250 dye. The dye serves to differentiate between holo- and apo-metalloprotein forms, as documented by electrophoretic mobility (CZE) and molecular docking (Autodock Vina) studies. The new HAC-2D MICS-BN-PAGE technique was followed by metal detection PAGE (to determine metal ions Cu^2+^, Fe^3+^, Co^3+^, Ni^2+^, Mn^2+^ and Cd^2+^) and by MALDI-TOF MS (for protein identification), allowing for the confirmation of bound metal ion and protein identities in biological samples. We expect this methodology will significantly contribute to “metallomics” to understand biological phenomena, including the discovery of new metalloproteins (and their functions) and the distribution of metal ions in real biological samples, just as conventional IEF/SDS-2D PAGE by O’Farrell^28^ contributed to the development of “proteomics”. Further studies to identify new separation mediator molecules for holo/apo recognition in PAGE (akin to the role of CBB-G 250 dye in these studies), are already underway in our laboratory.

## Methods

### Chemicals and regents

All reagents used were analytical or electrophoresis grade. Acrylamide (AA) was purchased from Tokyo Kasei (Tokyo, Japan). *N*,*N*′-methylenebisacrylamide (Bis) and CBB G-250 were purchased from Wako Pure Chemicals Industries (Osaka, Japan). Holo- and apo-human transferrin (Tf) were purchased from Merck (Darmstadt, Germany), human ceruloplasmin (Cp) was purchased from Sigma-Aldrich (St. Louis, USA) and Cu/Zn type superoxide dismutase from bovine erythrocytes (SOD) was purchased from Wako. Extracts of the soluble fraction from *Rubrivivax gelatinosus* (*R. gelatinosus*) were obtained from cells grown in malate medium at 30 °C under microaerobiosis conditions in the presence of 1.2 mM CuSO_4_. Cells were harvested after 48 h growth and the total soluble fraction was extracted as described by Durand *et al*.^22^. (see Supplemtantary Information for detail sample preparation procesure)

### Apparatus for PAGE

A PROTEAN® II xi Cell and PowerPacTM HV Power Supply (Bio-rad Laboratories, Hercules, USA) were employed for PAGE experiments. A thermostat water bath (LTB-125, AS ONE, Osaka, Japan) and an incubator (MIR-252, SANYO Electric, Osaka, Japan) were employed for controlling the temperature of the slab gels. A Printgraph (AR-9633FXCF-U, ATTO, Tokyo, Japan) and Safe Imager (*λ*_*ex*_ = 480 nm; Invitrogen, Carlsbad, USA) were used for fluorescence detection of metal complexes in metal detection PAGE.

### Procedure for MICS-BN-PAGE

The first and second stages of MICS-BN-PAGE were conducted according to our previous work^21^. Briefly, a slab gel composed of a separation gel (10% T, 2.7% C AA/BIS, 0.19 M Tris-HCl, pH 8.8, 20 µM EDTA) and a stacking gel (4% T, 2.7% C AA/BIS, 63 mM Tris-HCl, pH 7.4, 20 µM EDTA) was prepared. The slab gel was conditioned by applying 600 V for 1 h, with an upper (6.3 mM Tris-HCl, pH 7.4, 20 µM TPEN, 40 mM NaCl) and lower (6.3 mM Tris-48 mM Gly, pH 8.6, 10 µM CyDTA) migration buffer solution, to sweep out contaminant metal ions from the stacking gel. After removing the migration buffer including TPEN from the gel, sample solutions containing 6.3% glycerol, 3.2 mM Tris-HCl (pH 7.4), and 3.0 mM CBB G-250, were injected into the wells. A voltage of 600 V was applied with an upper migration buffer solution of Tris-Gly (6.3–48 mM, pH 8.6) without TPEN, until all sample solutions migrated into a stacking gel. Then, the upper solution was exchanged to a Tris-Gly (6.4–48 mM, pH 8.6) with 20 µM TPEN buffer solution, followed by concentration and separation by applying 900 V for 2 h. During all electrophoresis runs, the temperature of the slab gel was kept at 273 K to slow down the dissociation process.

### Procedure for HAC-2D MICS-BN-PAGE

After the first PAGE stage, the separation gel was cut along each lane, followed by application of the holo/apo conversion process prior to initiation of the second PAGE stage. Holo/apo conversion was performed in three steps. First, the gel lane was slowly shaken in a solution of 0.1 M HCl, 0.1 mM EDTA and 0.1% CBB G-250 for 25 minutes to dissociate metal ions from holo-metalloproteins. Second, the gel was shaken in a solution containing 0.13 M acetic acid buffer (pH 5), 0.1 mM EDTA and 0.1% CBB G-250 for 5 minutes to form metal complexes with EDTA, but not with apo-metalloproteins. Finally, the gel lane was shaken in a solution of 63 mM Tris-HCl (pH 7.4) and 0.1 mM EDTA for 5 minutes to establish the appropriate pH in the gel and to remove excess CBB dye. After the prepared gel lane was layered on the second slab gel, a metal-free 1% agarose gel was used to fill in the space between the first and second gels to prevent diffusion of proteins. Electrophoresis separation followed (with an applied voltage of 700 V for 2 h). This entire process constitutes the 2D MICS-BN-PAGE method, and the resulting gel was stained for detection of proteins (using silver or CBB staining).

### Identification of metalloprotein

After HAC-2D MICS-BN-PAGE, silver or CBB staining was conducted for the slab gel to detect the spots of isolated metalloprotein that had migrated off the diagonal line. The spots cut from the resulting slab gel were then subjected to in-gel tryptic digestion using the In-Gel Tryptic Digestion Kit (Thermo Fisher Scientific, Waltham, USA), according to the kit protocol. Digested sample solutions were desalted and concentrated by GL-TipTM SDB (GL Sciences, Tokyo, Japan) prior to being subjected to MALDI-TOF MS. For MALDI-TOF MS measurements, *α*-cyano-4-hydroxycinnamic acid (HCCA, Wako; for proteome) was employed as the matrix. As a calibration standard, a Peptide Calibration Standard II solution (Bruker Daltonics, Billerica, USA) was employed. The MALDI-TOF MS measurements by Autoflex III (Bruker Daltonics) were conducted using the reflector positive method (with molecular weight range 1–5 kDa). The protein was identified by peptide fingerprinting using publicly available databases (MASCOT, MatixScience).

### Capillary electrophoresis

The CZE separation was performed using a P/ACE MDQ CE-UV system (Beckman Coulter, Brea, USA), equipped with a 50 µm ID × 50.2 cm total-length fused-silica capillary (40 cm effective length) (GL Sciences). The separation buffer solution was composed of 30 mM Tris-HCl (pH 8.3). A sample solution of 10 µM holo- or apo-Tf, 30 mM Tris-HCl (pH 7.0) with the addition of 5 mM CBB G-250 was prepared. The sample solution was incubated for 1 h at 298 K. Typical hydrodynamic injection volume was 15 nL, and a 20 kV voltage was applied for CZE runs at a constant temperature of 298 K. The UV detector monitored absorbance at 195 and 580 nm (corresponding to the maximum absorption wavelengths of proteins and CBB G-250 dye).

### Molecular docking simulation by autodock Vina (version 1.1.2)

The crystal structure data of holo- and apo-Tf were obtained from RCSB Protein Data Bank7 (3V83 and 2HAU for holo- and apo-Tf, respectively). The PDB files were preprocessed and output as PDBQT files using AutoDock Tools 1.5.6 program. The steric structure of CBB G-250 was modeled using ChemBio 3D (version 14.0, PerkinElmer, Waltham, USA) and the optimized structure was calculated using molecular mechanics employing the MM2 parameter, followed by semi-empirical molecular orbital calculations using an AM1 basis set.

For flexible molecular docking simulations, AutoDock Vina software (version 1.1.2) was used. All 16 single bonds in the CBB G-250 structure were set as rotatable for the docking calculations. Exhaustive search procedure using the grid box size set to envelope the whole Tf structure was repeated twenty times to find most of the strong binding sites. After this procedure, a calculation using a smaller grid box was conducted to more precisely discover weaker binding sites (typical grid box size was set at 50 Å × 50 Å × 50 Å) for the whole surface of the protein. After searching binding sites (binding energy < −4.0 kcal/mol), each binding site was simulated to obtain more accurate coordination and energy using a smaller grid box (typically, x × y × z = 20 Å × 20 Å × 20 Å).

The detailed experimental conditions for PAGE, CZE and docking simulation are described in Supplementary Information.

## Supplementary information

Supplementary Information
