# Supplementary material for: Two-Dimensional Polyacrylamide Gel Electrophoresis for Metalloprotein Analysis Based on Differential Chemical Structure Recognition by CBB Dye
Source: Sci Rep. 2019 Jul 22;9:10566. doi: 10.1038/s41598-019-46955-6 (PMC6646366; doi:10.1038/s41598-019-46955-6)
Supplement: Supplementary file 1 — Supplementary Information [file 41598_2019_46955_MOESM1_ESM.docx]

Supplementary Information

**Two-Dimensional Polyacrylamide Gel Electrophoresis for Metalloprotein Analysis Based on Differential Chemical Structure Recognition by CBB Dye**

Junko Ishikawa, Akinori Maeshima, Allyson Mellinger, Anne Durand, Marie-Line Bourbon, Daichi Higo, Christa L. Colyer, Masami Shibukawa, Soufian Ouchane,Shingo Saito*

**Table of Contents**

**1. Detailed experimental procedures**

1-1. Chemicals and apparatus

1-2. MICS-BN-PAGE

1-3. Holo/Apo conversion two-dimensional MICS-BN-PAGE

1-4. Metal detection PAGE

1-5. Identification of metalloproteins

1-6. Capillary zone electrophoresis (CZE)

1-7. Molecular docking simulation by AutoDock Vina (version 1.1.2)

**2. Results**

**Table S1** Amino acid sequence fragments of CopI protein simulated by Biotools.

**Table S2** The binding sites in holo-Tf and the contacted amino acid residues in each site.

**Table S3** The binding sites in apo-Tf and the contacted amino acid residues in each site.

**Fig. S1** Typical electropherograms for BN-PAGE without MICS mode and MICS-BN-PAGE of standard protein samples of holo- and apo-transferrin, and concentrations of contaminant Cu^2+^ ions and Fe^3+^ ions in the separation gel of the BN-PAGE-without-MICS mode before separation.

**Fig.S2** Full-size versions of electropherograms represented in Fig. 2 Panel III and Fig. S5.

**Fig. S3** HAC-2D MICS-BN-PAGE/metal detection PAGE for a standard human serum sample.

**Fig. S4** Full-size versions of electropherograms represented in Fig. S3.

**Fig. S5** 2D MICS-BN-PAGE without holo/apo conversion between the first and the second PAGE.

**Fig. S6** Full-size version of electropherogram of 1D MICS-BN-PAGE represented in Fig. S5.

**Fig. S7** MALDI-TOF MS spectrum of the off-diagonal spot in HAC-2D MICS-BN-PAGE for a soluble fraction obtained from *R. gelatinosus* cells grown in the presence of 1.2 mM Cu^2+^.

**Fig. S8** Full-size versions of electropherograms represented in Fig. 3

**Fig. S9** Binding modes of the CBB-G250 molecule bound with holo-Tf and apo-Tf simulated using AutoDock Vina.

**Fig. S10** An example of the binding mode between apo-Tf and CBB-G 250, with −9.34kcal/mol binding affinity.

**4. References**

**1. Detailed experimental procedures:**

**1-1. Chemicals, protein samples, and apparatus**

**1-1-1. Chemicals**

All reagents used were analytical or electrophoresis grade. For polyacrylamide gel preparation, monomer stock solutions were first prepared by dissolving an adequate amount of acrylamide (AA, Tokyo Kasei, Tokyo, Japan; >98% purity) and *N,N’*-methylenebisacrylamide (Bis, Wako Pure Chemicals Industries, Osaka, Japan; 99% purity) into ultrapure water (produced by MilliQ Integral3, Merck Millipore, Burlington, USA) to a concentration of 60% T and 2.7% C, respectively. Aqueous solutions of 10% (w/v) ammonium persulfate (APS, Kishida Chemical, Osaka, Japan) and *N,N,N’,N’*-tetramethylethylenediamine (TEMED, Wako; > 99% purity) used as a polymerization initiator were prepared at the time of use. Stock solutions of tris(hydroxymethyl)aminomethane (Tris)-HCl and tris-glycine pH buffer were prepared by dissolving an adequate amount of Tris (Wako) and glycine (Kanto Chemical, Tokyo, Japan) powder in ultrapure water with the addition of diluted hydrochloric acid (Wako) to obtain the desired pH (as measured by pH meter model 9618S, HORIBA, Kyoto, Japan). A stock solution of acetate pH buffer was prepared by diluting an adequate volume of acetic acid in ultrapure water with the addition of 3 M sodium hydroxide solution (Kanto Chemical; ultrapure) to adjust to pH 5. Glycerol (Wako) and CBB G-250 (electrophoresis grade) (Wako) were employed as additives of the sample solutions.

Chelating agents for the MICS technique, including disodium ethylenediamine-*N,N,N’,N’*-tetraacetate dehydrate (EDTA, >99.5% purity), *N,N,N’,N’*-tetrakis(2-pyridylmethyl)ethylenediamine (TPEN, >97% purity), and trans-1,2-diaminocyclohexane-*N,N,N’,N’*-tetraacetic acid monohydrate (CyDTA), were purchased from Dojindo (Kumamoto, Japan).

**1-1-2. Protein samples**

Proteins (holo- and apo-human transferrin (Tf, Merck, Darmstadt, Germany; >95% purity), human ceruloplasmin (Cp, Sigma-Aldrich, St. Louis, USA), and Cu/Zn type superoxide dismutase from bovine erythrocytes (SOD, Wako)) were dissolved in ultrapure water to the desired concentration.

Total soluble fraction from *Rubrivivax gelatinosus* (*R. gelatinosus*) was obtained from cells grown in malate medium at 30°C under microaerobiosis conditions (100 mL flasks filled with 100 mL medium) in the presence of 1.2 mM CuSO_4_. Cells (OD at 680 nm = 0.55) were harvested by centrifugation (6,000 *g* for 20 min at 4°C) after 48 h growth, the cell pellet was washed 2 times with phosphate buffer 0.05 M (pH 7.5) and suspended in 20 mL of the same buffer containing 1 mM phenylmethylsulfonyl fluoride and DNase (25 µg/mL). Cells were lysed by two passages through a French press cell at 1000 p.s.i, and the extract was clarified by centrifugation (15,000 *g* for 30 min at 4°C). The supernatant was further centrifuged (117,000 *g* at 4°c for 1.5 h), and the resulting supernatant (constituting the total soluble fraction) was flash frozen in liquid nitrogen and stored at -80°C. Protein concentration was determined using the bicinchoninic acid assay (Sigma) using bovine serum albumin as a standard.

**1-1-3. Apparatus**

A PROTEAN^®^ II xi Cell (inner plates; 20 × 20 cm, outer plates; 22.3 × 20 cm, spacers; 0.5 or 1.0 mm thick) and PowerPac^TM^ HV Power Supply (Bio-rad Laboratories, Hercules, USA) were employed for PAGE experiments. A thermostat water bath (LTB-125, AS ONE, Osaka, Japan) and an incubator (MIR-252, SANYO Electric, Osaka, Japan) were employed for controlling the temperature of the slab gels. A Printgraph (AR-9633FXCF-U, ATTO, Tokyo, Japan) and Safe Imager (*λ*_ex_ = 480 nm; Invitrogen, Carlsbad, USA) were used for fluorescence detection of metal complexes in metal detection PAGE.

**1-2. MICS-BN-PAGE**

The first and second MICS-BN-PAGE stages were conducted according to our previous work^1^. A slab gel composed of a separation gel (10% T, 2.7% C AA/BIS, 0.19 M Tris-HCl, 20 µM EDTA, pH 8.8 at 273 K) and a stacking gel (4% T, 2.7% C AA/BIS, 63 mM Tris-HCl, 20 µM EDTA, pH 7.4 at 273 K) were prepared. Before sample injection, the gel was conditioned by applying 600 V for one hour with an upper (6.3 mM Tris-HCl, 40 mM NaCl, 20 µM TPEN, pH 7.4 at 273 K) and lower (6.3 mM Tris-48 mM Gly, 10 µM CyDTA, pH 8.6 at room temperature) migration buffer solution, to sweep out contaminant metal ions from the stacking gel toward to the negative and positive poles. Samples were prepared by mixing solutions of 6.3% (v/v) glycerol, 3.2 mM Tris-HCl (pH 7.4 at 273 K) and 3.0 mM CBB G-250. The prepared sample solution was injected into the wells after careful rinsing to remove the migration buffer, including TPEN. The gel and the upper solution was exchanged with a Tris-Gly (6.3 mM-48 mM, pH 8.6 at room temperature) buffer solution without TPEN to avoid ligand-exchange reaction with holo-metalloproteins. A voltage of 600 V was applied until all sample solutions migrated into a stacking gel. Then the upper solution was exchanged with a Tris-Gly (6.3 mM-48 mM, pH 8.6 at room temperature) – 20 µM TPEN buffer solution, followed by concentration and separation by applying 900 V for 1 hour. During all electrophoresis runs, the apparatus was cooled to keep the temperature of the slab gel constant at 273 K to reduce any dissociation processes. After the first PAGE dimension, the separation gel was cut along each lane and was then subjected to holo/apo conversion 2D MICS-BN-PAGE (lane A) or metal detection PAGE (lane B). After the first and second stages of MICS-BN-PAGE, the protein bands were visualized with silver staining^2^ or CBB R-250 staining (electrophoresis grade; Wako) ^3^.

**1-3. Holo/Apo Conversion Two-Dimensional MICS-BN-PAGE**

For isolation of holo-metalloproteins in the original sample, holo/apo conversion 2D MICS-BN-PAGE was conducted as follows. This chemical conversion from holo-metalloproteins to apo-metalloproteins was conducted via a three-step process conducted prior to the second stage of MICS-BN-PAGE. First, lane A obtained in the first MICS-BN-PAGE stage was slowly shaken in a solution of 0.1 M HCl, 0.1 mM EDTA and 0.1% (w/v) CBB G-250 for 25 minutes in order to dissociate metal ions from holo-metalloproteins. Second, lane A was shaken in a solution of 0.13 M acetic acid buffer (pH 5), 0.1 mM EDTA and 0.1% (w/v) CBB G-250 for 5 minutes to form metal complexes with EDTA, but not with apo-metalloproteins. Finally, lane A was shaken in a solution of 63 mM Tris-HCl, pH 7.4 at 273 K, 0.1 mM EDTA for 5 minutes to establish the appropriate pH in the lane and to remove excess CBB dye. After this, lane A was layered on the second slab gel (already conditioned to remove contaminant metal ions, see section 1-2), and a metal-free 1% agarose gel (Nacalai Tesque, Kyoto, Japan) was used to fill in the space between the first and second gels in order to avoid the diffusion of proteins during the electrophoresis run. The metal-free filler gel was first purified by MICS mode to remove any contaminant metal ions before use. The voltage was then applied at 700 V for 1 hour to obtain the HAC-2D MICS-BN-PAGE electropherogram. To determine the concentration of CopI bound with Cu ion (holo-CopI), the gel lane obtained in the first MICS-BN-PAGE stage was cut at 5 mm intervals and then subjected to SDS-PAGE, in which the standard Laemmli’s method was employed^4^. Judging from the intensities of CopI bands visualized by CBB R-250 staining, the amount of CopI (µg) was determined by means of a calibration curve using bovine serum albumin as a standard.

The procedure for purification of the agarose gel is described briefly as follows. The agarose gel (1% (w/v) agarose, 63 mM Tris-HCl, 20 µM EDTA, pH 7.4 at 273 K) was layered on the acrylamide gel (4% T, 2.7% C AA/BIS, 63 mM Tris-HCl, 20 µM EDTA, pH 7.4 at 273 K), the role of which was to prevent the agarose gel from slipping off the glass plate. The gel was conditioned by applying 300 V for 2 hours with an upper (6.3 mM Tris-HCl, pH 7.4, 20 µM TPEN, 40 mM NaCl) and lower (6.3 mM Tris-48 mM Gly, pH 8.6, 10 µM CyDTA) migration buffer solution. During electrophoresis runs, the agarose gel plate was cooled to 283 K. The metal-free agarose gel thus obtained was cut into 2.5 cm×16 cm pieces, and then stored in metal-free PTFE bottles in the refrigerator. When using the metal-free agarose gel, the gel was liquefied by heating at 363 K.

**1-4. Metal Detection PAGE**

Metal detection PAGE was conducted according to our previous work^1^. A separation gel (30% T, 2.7% C AA/BIS, 94 mM Tris-48 mM Gly, pH 9.4 at room temperature) and stacking gel (17% T, 2.7% C AA/BIS, 94 mM Tris-HCl, pH 8.8 at 273 K) were prepared for metal detection PAGE. Lane B obtained from the first MICS-BN-PAGE gel was cut at 5 mm intervals prior to acid extraction of metal ions (which were bound to proteins), by soaking the gel fractions in 100 µL of 0.2 M HCl (TAMAPURE-AA-10, Tama Chemicals, Kanagawa, Tokyo; impurity <10 ppb) solution for 2 h. The samples subjected to metal detection PAGE were prepared by mixing a solution of 25 µL (50% (v/v) in total volume) eluted sample solution, 1 µM FTC-ABDOTA (a fluorescent probe for detection of Fe^3+^, Cu^2+^, Ni^2+^, Co^3+^, Mn^2+^, Cd^2+^)^1,5^, and 0.22 M acetic acid buffer (pH 5). Finally, the pH of the mixture was adjusted to 8 with 0.15 M Tris-HCl (pH 10) in order to avoid aggregation of CBB G-250 bound with proteins in the first MICS-BN-PAGE stage (which would cause negative effects for the detection of metal ions in the separation gel). The prepared samples were injected into wells and then automatically concentrated and separated by an applied voltage of 1200 V for 2 hours. The slab gel was cooled to 288 K during separation. After separation, the bands of metal complexes were detected by fluorescence detection using a CCD camera with a Safe Imager. The gel images were analyzed by using CS Analyzer 3 software (ATTO).

**1-5. Identification of Metalloprotein**

After HAC-2D MICS-BN-PAGE, silver or CBB staining was conducted for the slab gel to detect the spots of isolated metalloprotein that had migrated off the diagonal line. The spots cut from the resulting slab gel were then subjected to in-gel tryptic digestion using the In-Gel Tryptic Digestion Kit (Thermo Fisher Scientific, Waltham, USA), according to the kit protocol. Digested sample solutions were desalted and concentrated by GL-Tip^TM^ SDB (GL Sciences, Tokyo, Japan) prior to being subjected to MALDI-TOF MS. Finally, the sample solutions were heated and dried using an evaporator (MV-100, TOMY SEIKO, Tokyo, Japan), followed by the addition of 5 µL of 80% (v/v) acetonitrile (Wako) and 0.1% (v/v) trifluoroacetic acid (TFA, Wako) solution.

For MALDI-TOF MS measurements, α-cyano-4-hydroxycinnamic acid (HCCA, Wako; for proteome) was employed as the matrix. HCCA was dissolved in TA solvent (acetonitrile:0.1% TFA was 1:2) up to saturation at room temperature aided by ultrasonication. A 1 µL portion of the matrix solution and a 1 µL portion of the sample solution were mixed prior to dropping onto the standard steel MALDI target plate (MTP 384 target plate ground steel TF, Bruker Daltonik GmbG, Bremen, Germany), followed by spontaneously drying the spots at room temperature. As a calibration standard, a Peptide Calibration Standard II solution (Bruker Daltonics, Billerica, USA) was dissolved in 0.1% TFA and applied onto the target plate. The MALDI-TOF MS measurements by Autoflex III (Bruker Daltonics) were conducted using the reflector positive method (with molecular weight range 1-5 kDa). The protein detected in the spot from HAC-2D MICS-BN-PAGE was identified by peptide fingerprinting using publicly available databases (MASCOT, MatixScience).

**1-6. Capillary Electrophoresis**

The capillary zone electrophoresis (CZE) separation was performed using a P/ACE MDQ CE-UV system (Beckman Coulter, Brea, USA), equipped with a 50 µm ID × 50.2 cm total-length fused-silica capillary (40 cm effective length) (GL Sciences). The capillary was rinsed sequentially with 0.1 M NaOH (15 minutes), pure water (15 minutes), and a separation buffer solution (15 minutes) by applying pressure (25 psi). The separation buffer solution was composed of 30 mM Tris-HCl (pH 8.3), which was the same as the separation gel buffer solution used for MICS-BN-PAGE. A sample solution of 10 µM holo- or apo-Tf, 30 mM Tris-HCl (pH 7.0) with the addition of 5 mM CBB G-250 was prepared, which was the same solution as used for the sample buffer solution and the stacking gel buffer solution in MICS-BN-PAGE. The sample solution was incubated for 1 h at 298 K. The typical hydrodynamic injection volume was 15 nL, and a 20 kV voltage was applied for CZE runs at a constant temperature of 298 K. The UV detector monitored absorbance at 195 and 580 nm (corresponding to the maximum absorption wavelengths of proteins and CBB G-250 dye, respectively).

**1-7. Molecular Docking Simulation by AutoDock Vina (version 1.1.2)**

The crystal structure data of holo- and apo-Tf were obtained from the RCSB Protein Data Bank^6^ (3V83 and 2HAU for holo- and apo-Tf, respectively). The PDB files were preprocessed and output as PDBQT files using AutoDock Tools 1.5.6 program. The steric structure of CBB G-250 was modeled using ChemBio 3D (version 14.0, PerkinElmer, Waltham, USA) and the optimized structure was calculated using molecular mechanics employing the MM2 parameter, followed by semi-empirical molecular orbital calculations using an AM1 basis set.

For flexible molecular docking simulations, AutoDock Vina software (version 1.1.2) was used. Recently, Wang et al. ^7^ reported that AutoDock Vina has high scoring power and intermediate sampling power relative to other widely-used docking programs. While all 16 single bonds in the CBB G-250 structure were set as rotatable for the docking calculations, it was also reported that the AutoDock Vina program was able to provide a high success rate for docking even with ~20 rotatable bonds in the calculation^7^. Although some of the most stable binding modes of CBB G-250 were found by exhaustive searching using the grid box size set to envelope the whole Tf structure, the random binding sites were found in repeated runs. In contrast to the usual use of this program to search for the most stable binding mode, the need to find many binding sites of CBB G-250 (including not only strong but also intermediate or weak binding interactions) was important to this work to simulate the mixture of Tf with a large excess of the dye. As such, the exhaustive search procedure using such a large grid box was repeated twenty times to find most of the strong binding sites (with the exhaustiveness and the num_mode parameters set at 10 and 20, respectively). After this procedure, a calculation using a smaller grid box was conducted to more precisely discover weaker binding sites (typical grid box size was set at 50 Å × 50 Å × 50 Å) for the whole surface of the protein.

After searching binding sites (binding energy < −4.0 kcal/mol), each binding site was simulated to obtain more accurate coordination and energy using a smaller grid box (typically, x × y × z = 20 Å × 20 Å × 20 Å). In this calculation, the exhaustiveness and the num_mode parameters were set to 50 and 5, respectively.

**Results and Discussion**

**2. Results**

**Table S1** Amino acid sequence fragments of CopI protein, simulated by Biotools.

| Mass | Amino acid sequence |
| --- | --- |
| 560.292 | GETVR |
| 790.409 | FVAANGGR |
| 841.386 | AHAQEMR |
| 937.436 | VVMSDTMR |
| 1018.545 | FDPATITVR |
| 1494.671 | AMPDMQHADPGAVR |
| 1543.825 | IEHEFVLGTTASLK |
| 1562.809 | VAAGASGEIVWQFTK |
| 2167.023 | AGSFEEFACLIPGHFEAGMVGK*^a^* |

*^a^* The fragment with m/z = 2225 in Fig. S7 included the 2167.023 fragment, as revealed by MS/MS analysis.

**Table S2** The binding sites in holo-Tf and the contacted amino acid residues in each site.*^a^*

*^a^* Residues highlighted in white, green, and red are hydrophobic, hydrophilic, and charged amino acids, respectively.

**Table S3** The binding sites in apo-Tf and the contacted amino acid residues in each site.*^a,b^*

*^a^* Residues highlighted in white, green, and red are hydrophobic, hydrophilic, and charged amino acids, respectively.*^b^* Site numbers in red text in the first column represent binding sites that were not observed in holo-Tf.


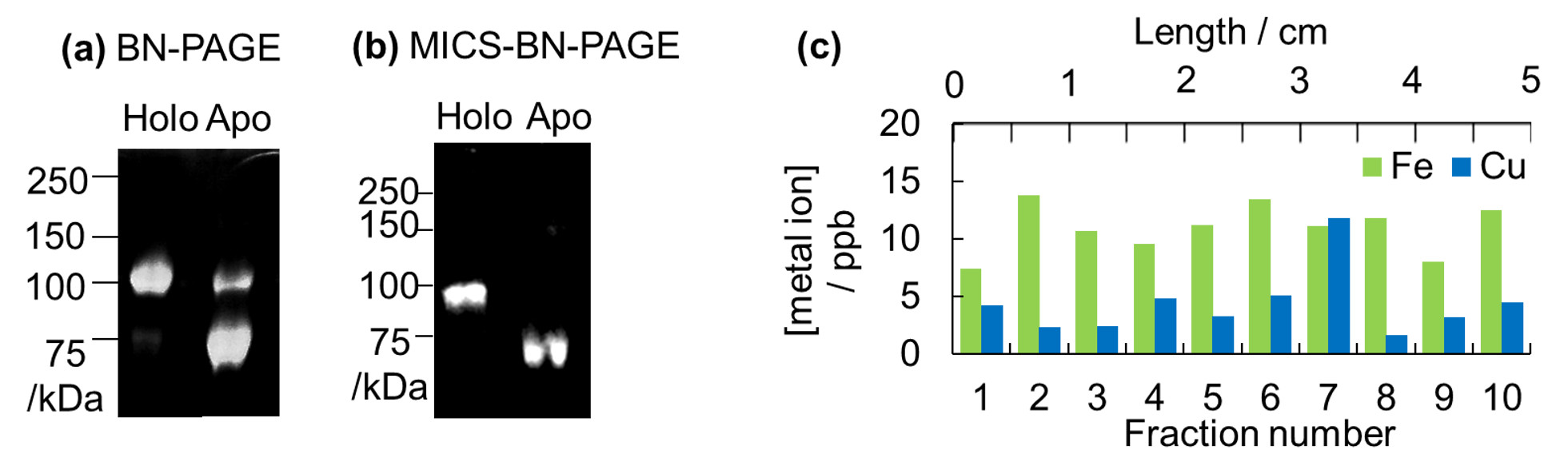


**Fig. S1** Typical electropherograms for BN-PAGE (a) and MICS-BN-PAGE (b) of standard protein samples of holo- and apo-transferrin (Tf) with CBB R-250 staining, and concentrations of contaminant Cu^2+^ ions (blue bars) and Fe^3+^ ions (green bars) in the separation gel of the BN-PAGE-without-MICS mode before separation (that is, blank concentrations in the separation gel) (c). In the sample (a) and (b), [Tf] = 2.5 µM; loaded volume = 10 µL. Two distinct bands were detected in conventional BN-PAGE (without MICS mode) for a “pure” apo-transferrin (Tf) sample (Fig. S1(a)), in which CBB G-250 dye serves as a weak denaturing agent. This is possibly due to the in-gel complexation of apo-metalloproteins with contaminant metal ions, since up to 10 ppb levels of contaminant metal ions (Fe^3+^ and Cu^2+^) existed in the whole gel fractions as seen in Fig. S1(c). Thus, without employing the MICS mode, contamination by trace metal ions is unavoidable, resulting in mis-identification of metalloproteins. The upper and lower bands were assigned as holo (Fe_2_-Tf) and apo-Tf, respectively. The Fe_2_-Tf species formed due to Fe contamination, while Fe^3+^ was detected only from the upper fraction (data not shown). Using the MICS mode, the upper band effectively disappeared in an apo-Tf sample, as shown in Fig. S1(b). That is, one band was successfully observed for each form of Tf (holo- and apo-) at different migration positions. As such, the MICS mode is one of the essential keys for accurate holo-metalloprotein analysis.

**
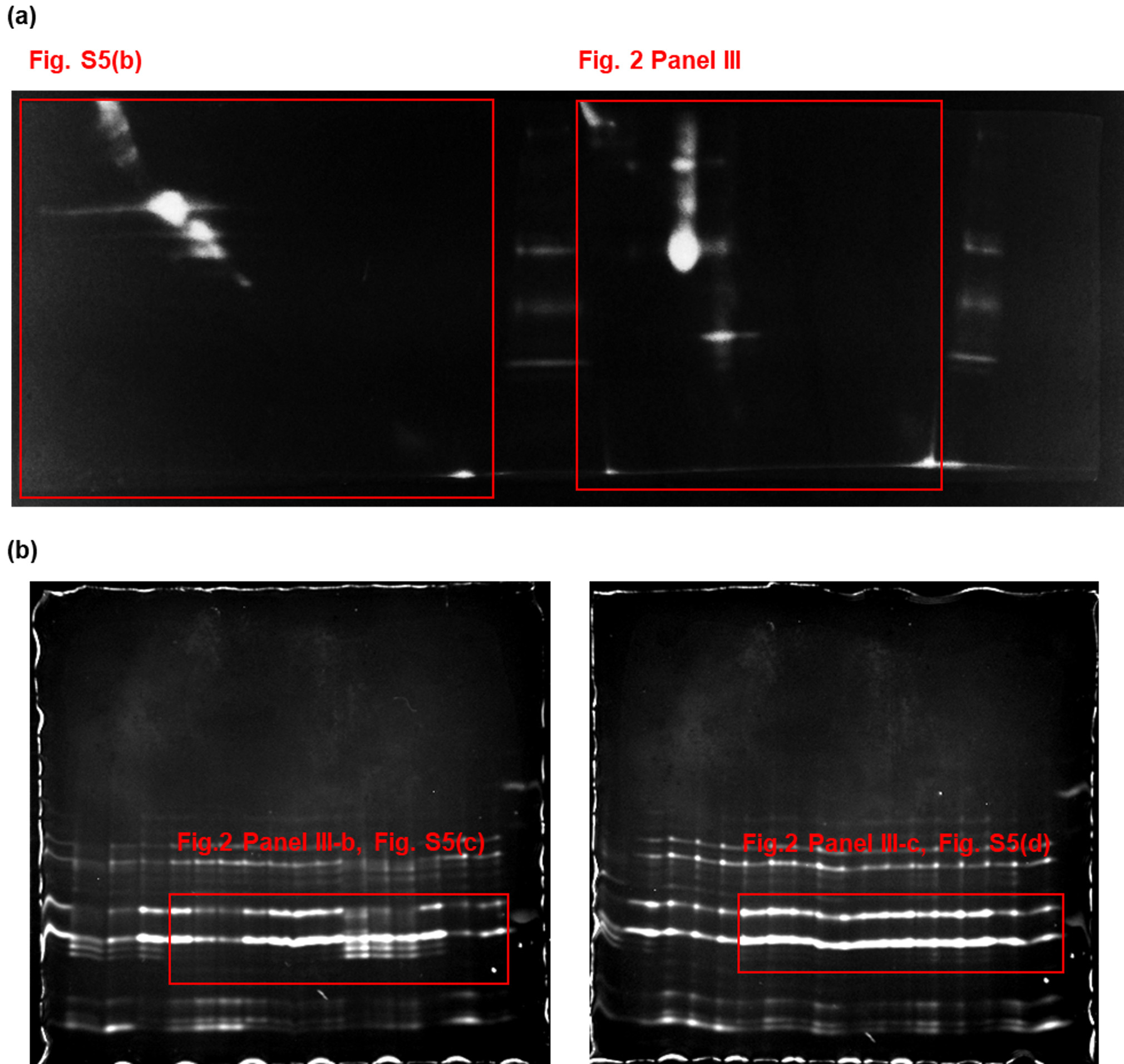
**

**Fig. S2** Full-size versions of electropherograms of HAC-2D MICS-BN-PAGE (a) and metal detection PAGE (b) represented in Fig. 2 Panel III and Fig. S5.


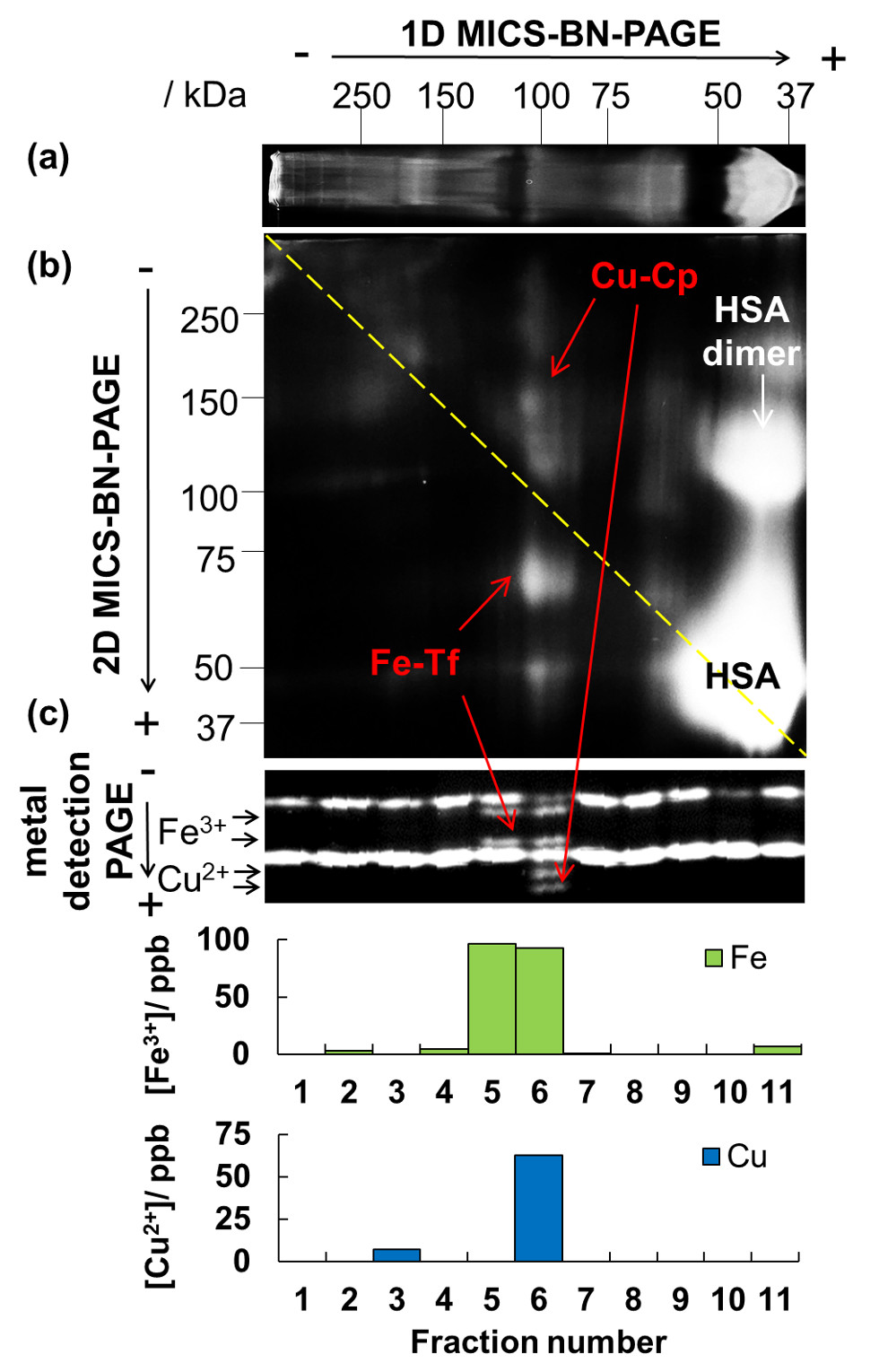


**Fig. S3** HAC-2D MICS-BN-PAGE/metal detection PAGE for a standard human serum sample, obtained from a 28-year old male. A separation gel with 8% T, 2.7% C was employed for the separation and silver staining was used for the detection. The human serum sample was diluted by 50% with sample buffer solution including CBB G-250. The loaded volume was 10 µL. Human serum albumin protein is identified as HSA in the figure. The full-size versions of electropherogram are depicted in Fig. S4.

**
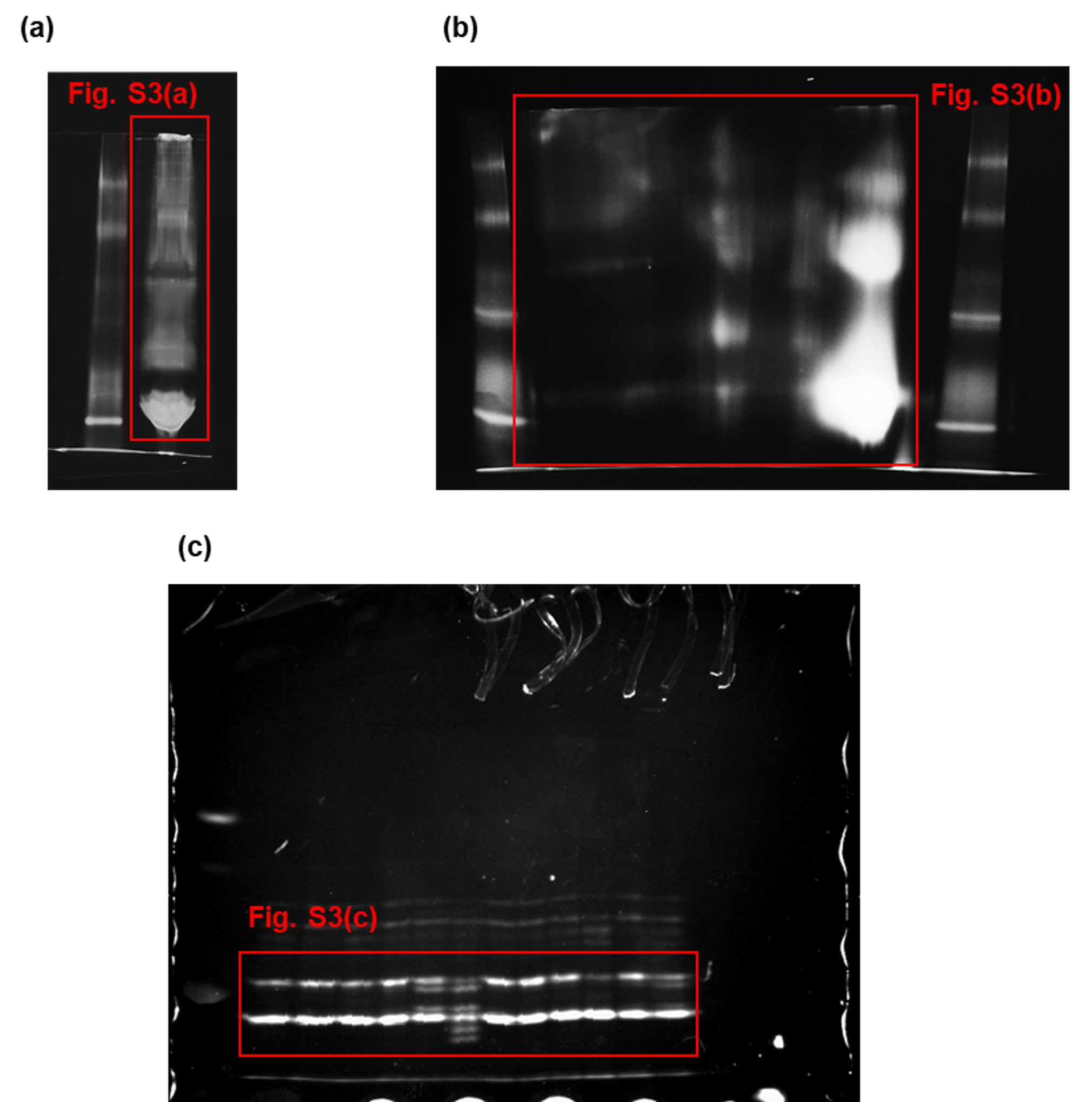
**

**Fig. S4** Full-size versions of electropherograms of 1D MICS-BN-PAGE(a), HAC-2D MICS-BN-PAGE (b) and metal detection PAGE (c) represented in Fig. S3.

　
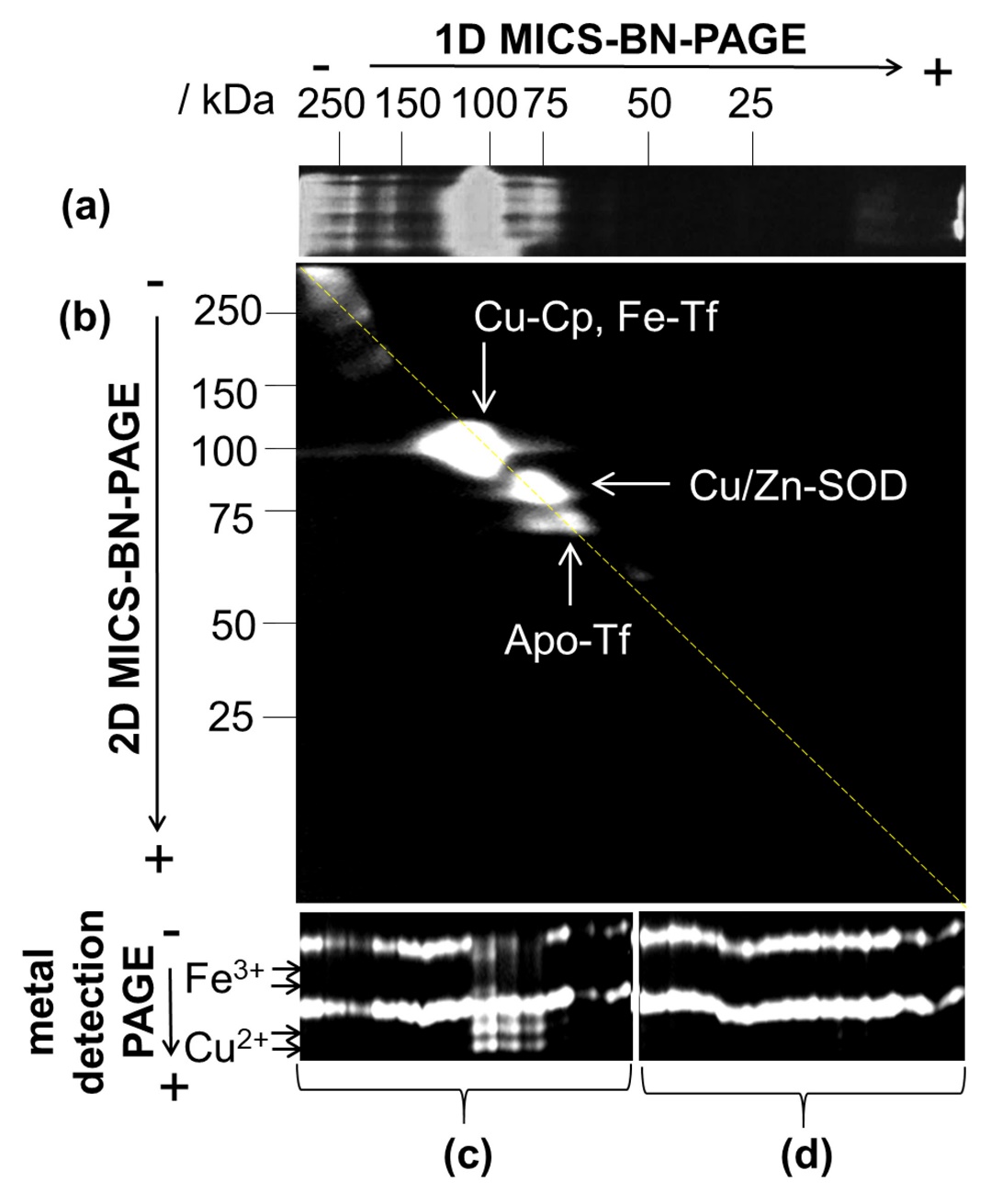


**Fig. S5** 2D MICS-BN-PAGE without holo/apo conversion between the first and the second PAGE. All proteins (holo-Cp, holo-Tf, apo-Tf and holo-SOD) migrated on the diagonal line with CBB R-250 staining. [holo-Cp] = 5 µM, [holo-Tf] = 10 µM, [apo-Tf] = 10 µM, [holo-SOD] = 25 µM; and loaded volume = 10 µL. Full-size versions of electropherograms presented in Fig. S5 are depicted in Fig. S2 and Fig. S6.

　
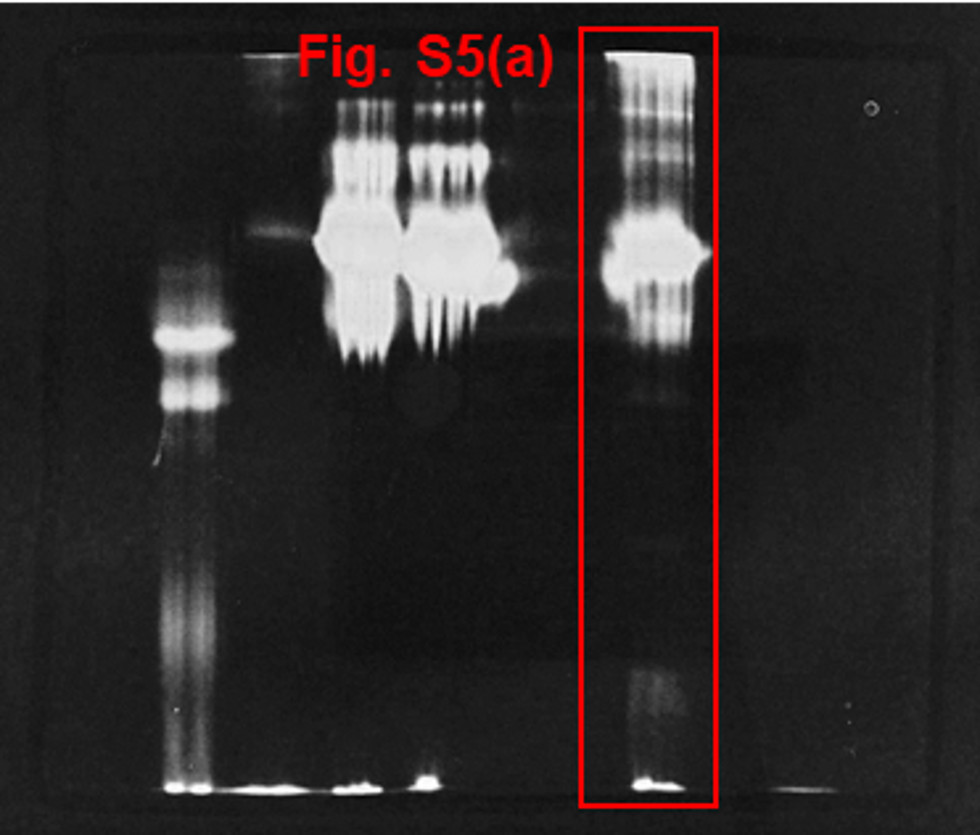


**Fig. S6** Full-size version of electropherogram of 1D MICS-BN-PAGE(a) represented in Fig. S5.


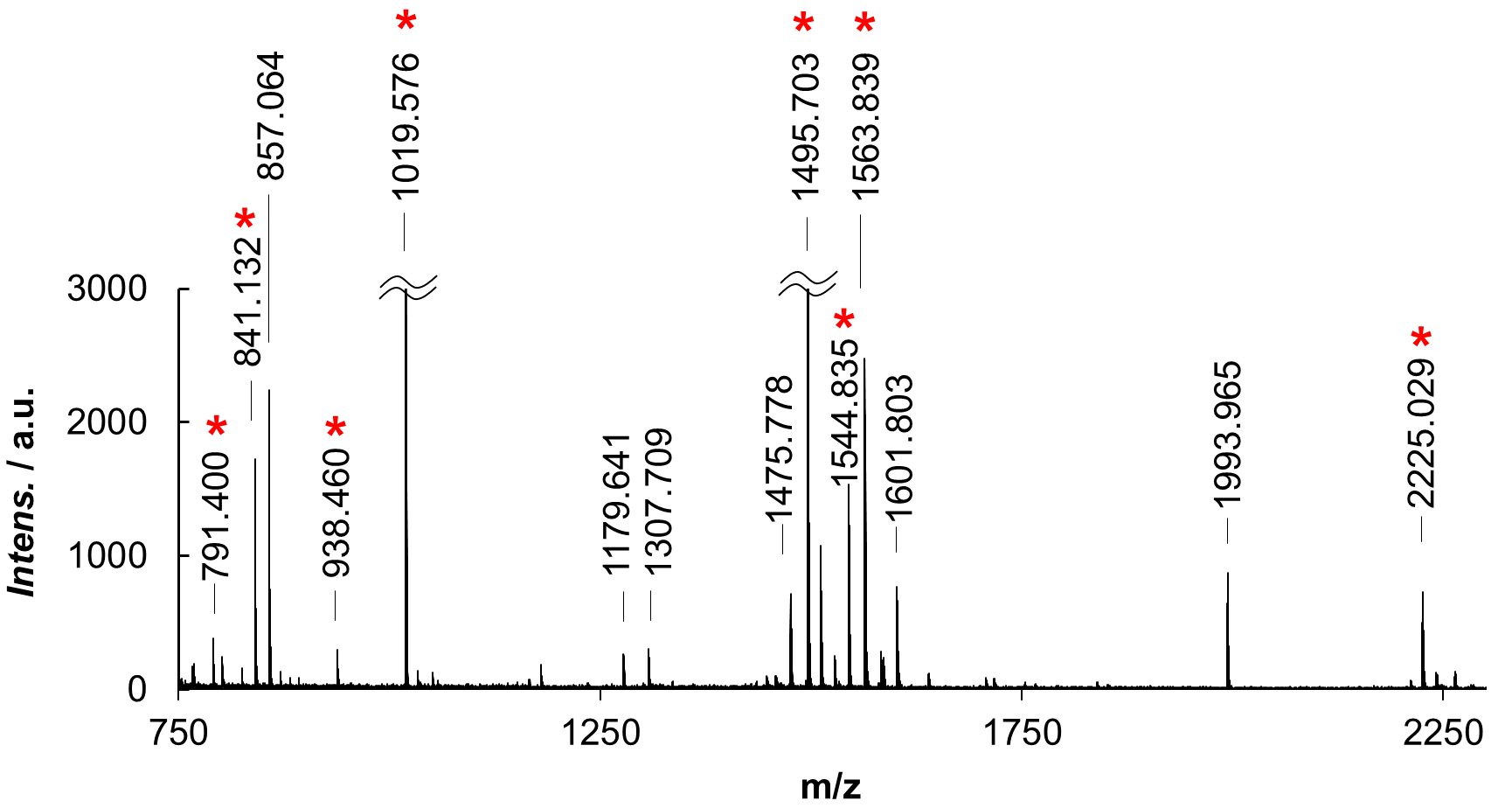


**Fig. S7** MALDI-TOF MS spectrum of the off-diagonal spot (indicated by the asterisk in Fig. 3(a)) in HAC-2D MICS-BN-PAGE for a soluble fraction obtained from *R. gelatinosus* cells grown in the presence of 1.2 mM Cu^2+^. Peaks marked by an asterisk represent fragment of CopI (Table S1).

**
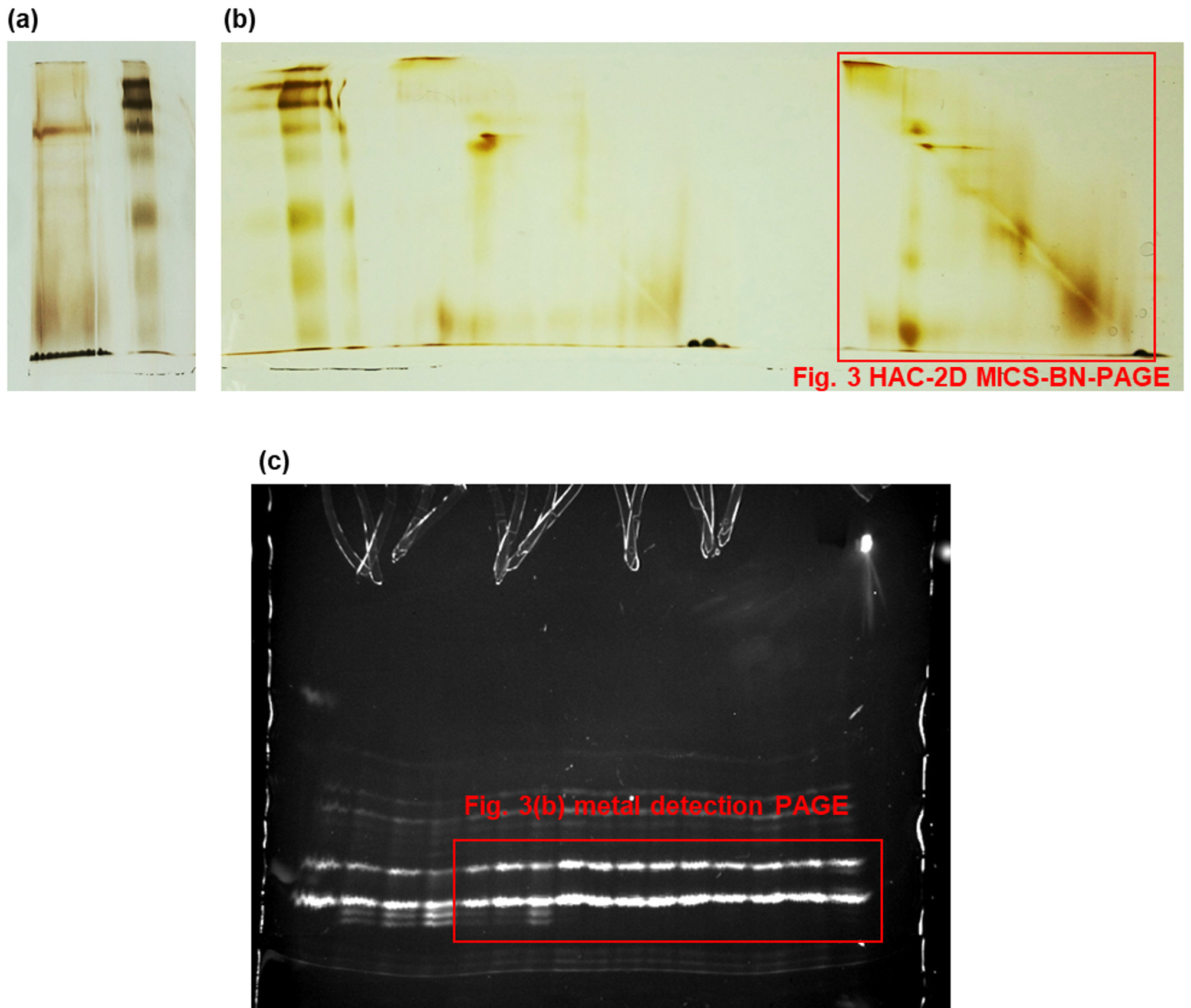
**

**Fig. S8** Full-size versions of electropherograms of 1D MICS-BN-PAGE(a), HAC-2D MICS-BN-PAGE (b) and metal detection PAGE (c) represented in Fig. 3.


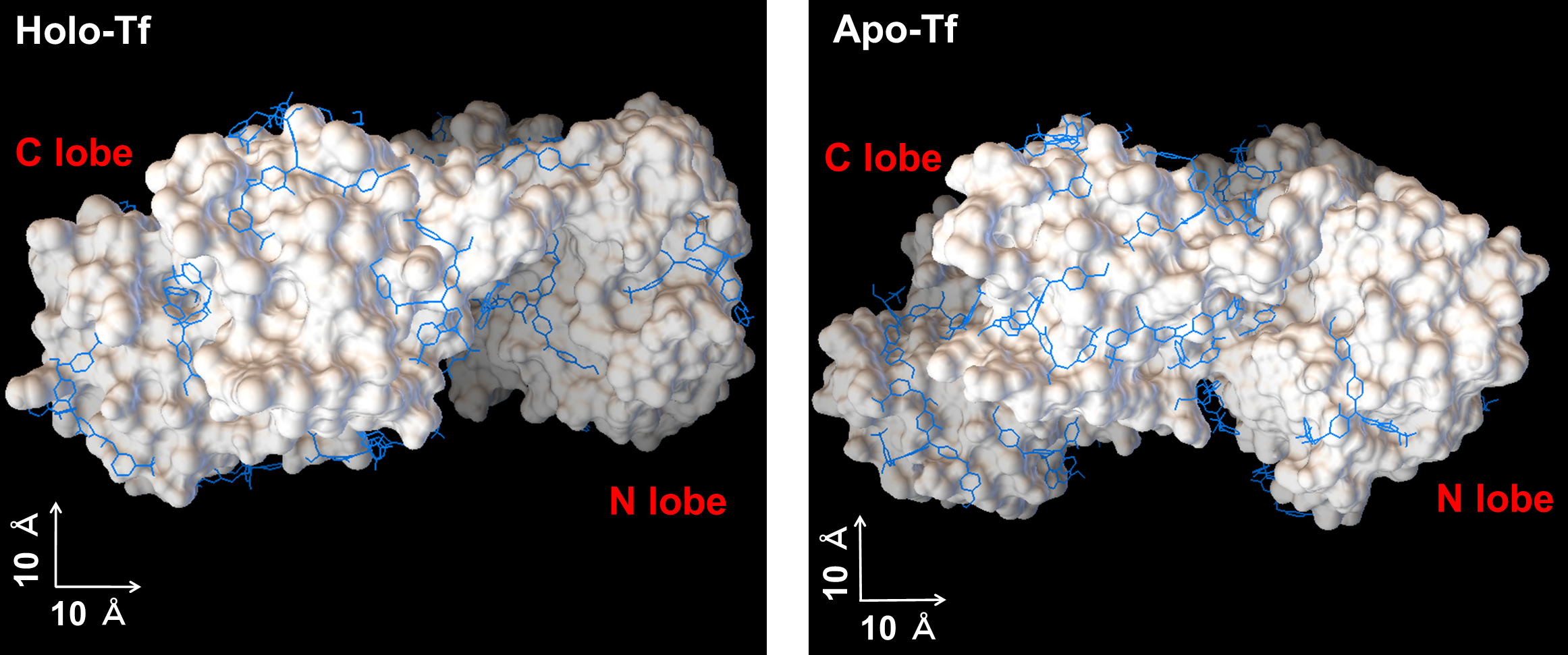


**Fig. S9** Binding modes of CBB G-250 molecules bound to holo-Tf (left) and apo-Tf (right), simulated using AutoDock Vina (affinity < −6.5 kcal/mol).


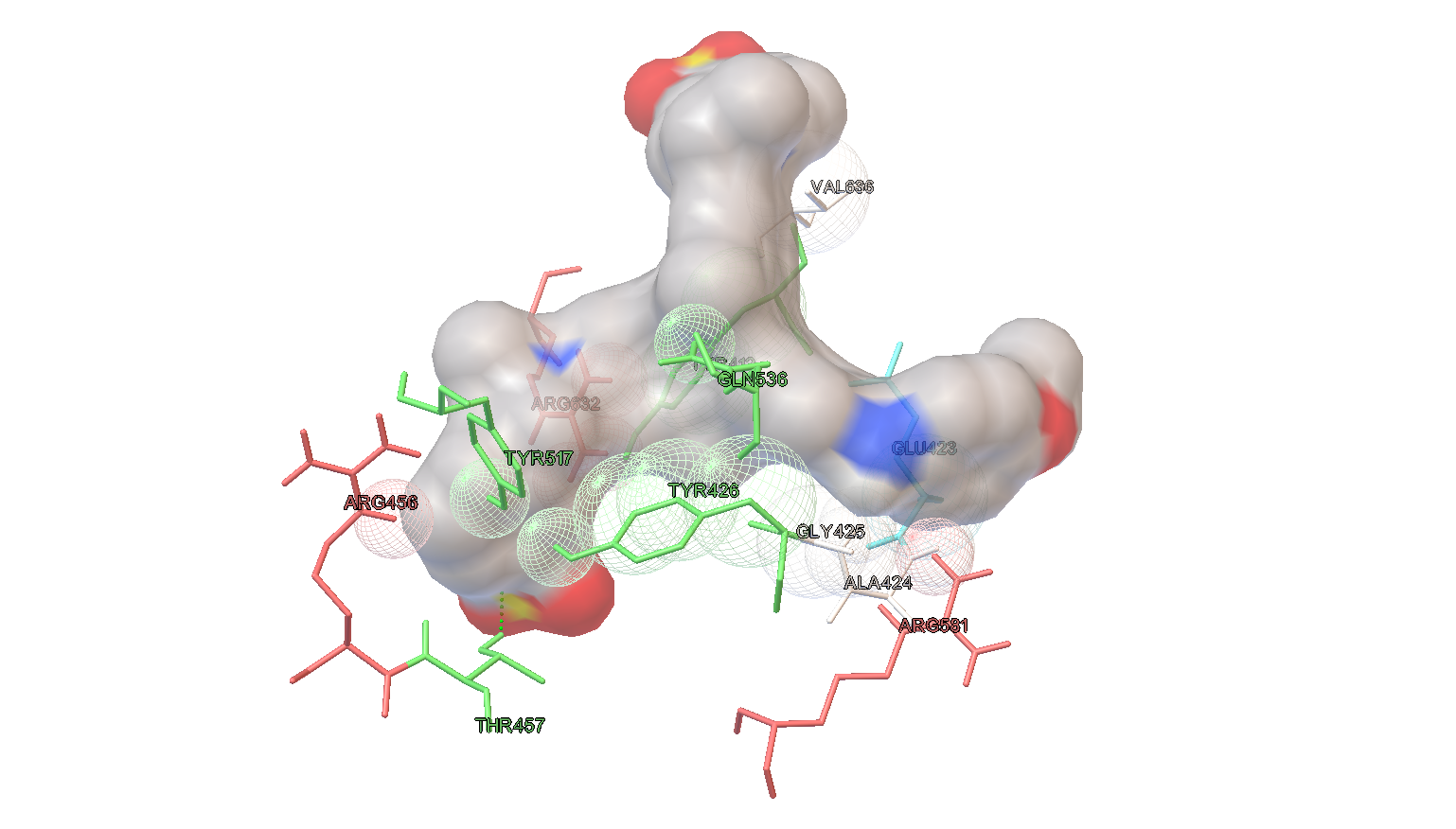


**Fig. S10**An example of the binding mode between apo-Tf and CBB-G 250, with -9.34 kcal/mol binding affinity. The molecule depicted by the CPK model is the CBB G-250 dye. Amino acid residues are grouped according to type: hydrophobic (white), hydrophilic (green), and charged (red). The atoms in contact with the molecular surface of the dye are depicted by mesh spheres. Hydrogen bonds are depicted by dashed lines.

**4. References**

(1) Saito, S. et al., Separation of metalloproteins using a novel metal ion contaminant sweeping technique and detection of protein-bound copper by a metal ion probe in polyacrylamide gel electrophoresis: distribution of copper in human serum. *Analyst* **138**, 6097-6105 (2013).

(2) Sinha, P., Poland, J., Schnölzer, M., Rabilloud, T., A new silver staining apparatus and procedure for matrix-assisted laser desorption/ionization-time of flight analysis of proteins after two-dimensional electrophoresis. *Proteomics* **1**, 835-840 (2001).

(3) He, F., Coomassie Blue Staining. *Bio-Protocol* **Bio101**, e78 (2001).

(4) Laemmli, K. U., Cleavage of Structural Proteins during the Assembly of the Head of Bacteriophage T4. *Nature* **227**, 680-685 (1970).

(5) Saito, S. et al., Ultrasensitive CE for heavy metal ions using the variations in the chemical structures formed from new octadentate fluorescent probes and cationic polymers. *Analyst* **136**, 2697-2705 (2011).

(6) https://www.rcsb.org/pdb/home/home.do

(7) Wang, Z. et al., Comprehensive evaluation of ten docking programs on a diverse set of protein-ligand complexes: the prediction accuracy of sampling power and scoring power. *Phys. Chem. Chem. Phys*. **18**, 12964-12975 (2016).
